# Objective greenness, connectedness to nature and sunlight levels towards perceived restorativeness in urban nature

**DOI:** 10.1038/s41598-023-45604-3

**Published:** 2023-10-24

**Authors:** Giuseppina Spano, Elisabetta Ricciardi, Annalisa Theodorou, Vincenzo Giannico, Alessandro Oronzo Caffò, Andrea Bosco, Giovanni Sanesi, Angelo Panno

**Affiliations:** 1 Department of Education, Psychology, Communication Sciences, University of Bari Aldo Moro, Via Scipione Crisanzio, 42 70122 Bari, Italy; 2 Department of Education, Experimental Psychology Laboratory, Roma Tre University, Rome, Italy; 3 Department of Soil, Plant and Food Sciences, University of Bari Aldo Moro, Bari, Italy; 4 Department of Human Science, Experimental and Applied Psychology Laboratory, European University of Rome, Rome, Italy

**Keywords:** Psychology, Environmental sciences

## Abstract

The beneficial effect of exposure to nature and immersion in natural environments on perceived well-being is well established. Nevertheless, we acknowledge an emerging need to disentangle the role of specific environmental features from individual factors that encourage a positive person-environment interaction. This study aimed at evaluating the associations between four buffer distances of greenness and dimensions of perceived restorativeness, with connectedness towards nature (CTN) as a confounder variable, in a sample of 312 visitors to a large urban park. Variables investigating ecosystem services (ES, e.g., thermal comfort) were included as covariates. Results revealed differentiated effects of greenness level, sunlight intensity, and connectedness to nature in the pathways towards dimensions of restorativeness. Greenness level at 300 m was associated with Fascination, Scope, and Being Away, while at 500 m was associated with Coherence, Scope, and Being Away. ES was found to be associated with Coherence, while CTN with the other three dimensions of restorativeness. The moderating effect of sunlight level in the relationship between NDVI buffer distances and the total score of perceived restorativeness was also confirmed. The present work is intended to offer insights on the interplay between environmental features and individual differences for implications in several contexts, including the opportunity to develop tailor-made planning for urban forestry.

## Introduction

A notable amount of evidence is available on the beneficial effect of direct contact with urban nature, such as parks, urban gardens, and public green and blue spaces, on health and wellbeing across the lifespan^1–5^.

Two of the most influential theoretical frameworks used to explain the link between exposure to natural environments and perceived well-being are the Attention Restoration Theory (ART)^6^ and the psychophysiological Stress Reduction Theory (SRT)^7^. ART states that natural environments possess a set of qualities that make them with a high restorative power for the recovering of mental fatigue and depleted cognitive resources, e.g., directed attention. An environment, to be restorative, should possess the ability to fascinate (“Fascination” dimension), to trigger a psychological distance from daily routine (“Being away” dimension), being compatible with the preferences and needs of the individual (“Scope” dimension), and provide a feeling of order and connection between the elements within it (“Coherence” dimension). Among the dimensions of perceived restorativeness, Coherence concerns the relationships between objects present in an environment, with an eye to the external world; on the contrary, the other three PRS dimensions concerns the internal world of the visitors within an environment, i.e., the feeling of fascination, scope, and being elsewhere in a psychological sense^6^. On the other hand, psychophysiological SRT argues that natural environments, unlike urban environments, promote physiological and psychological recovery from stress in individuals experiencing acute stress, through the increase of positive emotions^7^.

With increasing urbanization, reaching distant and wild natural environments can be difficult. For this reason, nature-based solutions within the city, including urban green spaces, are of paramount importance for supporting the relationship between nature and human well-being.

Parks represent a valuable source of ecosystem services, such as clean air, climate regulation, soil stabilisation, noise protection, for both ecological and human health, by providing biodiversity, protecting from heat waves and Heat Island Effect, mitigating air pollution and noise in certain district area, promoting physical activity, reducing perceived stress, improving social interaction, perceived quality of life, and perceived restorativeness^8–12^. Use of urban nature-based spaces encourage the development of a sense of connection with nature, and accordingly, a more favourable attitude towards its protection, pro-environmental attitude, and sense of place^13,14^. Furthermore, a visible increase in the use and in the perceived importance of urban green spaces by visitors, especially in large urban areas, has been recently observed as a consequence of the pandemic-related measures of containment of infection^15–18^.

On the basis of the well-established assumption of “nature is good”, a growing literature is focusing on the drivers and the underlying mechanisms within the relationship between the use of greenspace and the derived physical and mental health benefits (e.g.,^19^).

Individual differences, such as dispositions, personality traits, and demographic characteristics, play a significant role in the relationship between natural environments and perceived well-being^20,21^. For example, connectedness to nature is often considered as a stable trait-like characteristic^22^ that pertains to individual differences, demonstrate to affect motivation, emotions and the perceived restorativeness related to experiences in nature (e.g.,^23–25^). Sense of connection to nature showed to be associated with the perceived restorativeness provided by a natural environment^26^. A recent work^27^ reported a significant association between nature exposure and quality of life, mediated by nature connectedness, nature restorativeness and stress level, with nature restorativeness predicted both by nature connectedness and nature exposure.

On the other side, evidence on the influence of natural elements measured by objective measures on well-being is currently inconsistent.

Reyes-Riveros et al*.*^28^ reviewed a great number of studies (*N* = 153) to identify the relationship between specific characteristics of a green space (i.e., structure, biodiversity, naturalness, and others) and well-being dimensions (i.e., health, security, good social relations, and freedom of choice and action). The review provided a summary of the specific effects on each category of green space’s characteristics on each well-being dimension. Structure (e.g., vegetation cover) and biodiversity (e.g., number and diversity of plant species) were rated as the most important in improving human well-being in all aspects, and especially in health. Moreover, structure, biodiversity, and naturalness positively impact the dimension of “health”, including stress reduction, mental health, and physical activity.

Perceived biodiversity of green and blue spaces and urban sites seems to impact on the perceived restorativeness of the area, for example in a sample of campus students^29,30^.

Most of the available studies with objective green measurements focused on the effects of the so-called “residential surrounding greenness” exposure, i.e., the amount of available greenspace within a radius of usually 300 or 500 m around a familiar space, such as home or school. The buffer approach is typically used for fixed places, such as home, school, and workplace, thus considering the effect of a long-term greenness exposure. This approach is well-established especially in the field of environmental epidemiology, investigating physical and mental health outcomes^31,32^. The protective effect of a long-term greenness exposure has been widely observed on a variety of health outcomes, including the most recent evidence on fetal growth^33^.

Evidence is available based on the restorative attributes in green spaces linked to predictors of well-being^34–36^; however, the use of greenness indicators, such as NDVI, assessed using remote sensing technique, for exploring the association with perceived psychological outcomes, is currently lacking.

Likewise, the effect of different levels of sunlight on perceived health and well-being within urban green space has been poorly investigated. Notable evidence on this topic is provided by the work of Beute and de Kort^37–39^. Lighting showed to be associated with stress levels, mood, and mental health^38^. Interestingly, authors found that a natural, bright, and sunny scene was preferable over alternatives based on three dimensions of naturalness (nature vs. urban), brightness (light vs. dark), and weather type (sunny vs. overcast)^39^. Combination of nature and sunlight was also found to be beneficial on affect and stress-related outcomes^37^. Recent studies explored the effect of outdoor lighting levels by using virtual reality as it allows to easily manipulate the lighting levels, simulating an outdoor environment. However, most of the studies on this topic concern the investigation of the effect of artificial light in urban contexts (e.g., in streets, squares, or urban parks) for design purposes^34–36^. Available evidence on the effect of simulated natural light exposure reported that outdoor natural light characteristics, such as brightness, may enhance the perception of restorativeness of an environment^37^. Furthermore, lighting may affect the visual aspect and attraction of an urban park^40,41^, which, we can speculate, could trigger the fascination effect as described by the ART. Conversely, a lack of lighting, for example in a dark forest, may elicit negative emotions, such as fear, and avoidance tendencies as responses^40,42^.

From this picture it emerges that the relationship between greenness exposure (vegetation coverage) and sunlight intensity is still unclear. Green areas provide shade, cooling, and ecosystem benefits, influenced by sunlight. This interaction impacts human well-being and urban planning, but it varies seasonally and by location. Balancing these factors is crucial for sustainable and liveable environments. Unfortunately, there are few available studies on the interaction effect of outdoor natural lighting variation on psychological outcomes.

In order to contribute to overcome the highlighted research gaps and following the theory of the biophilic design^43^ which identified environmental elements in built environment that foster a positive human-nature connection, the aim of the present study is twofold:

1. To test the impact of four different levels of objective short-term greenness exposure (as a visitor) on each of the four dimensions of perceived restorativeness (i.e., fascination, being away, coherence, and scope), with connectedness towards nature as a confounder variable, with the advantage of testing it in a large urban park, without excessive intrusion of so-called gray elements (e.g., buildings, roads, etc.);
2. To investigate the moderating effect of objectively measured sunlight intensity (i.e., high vs. low intensity) in the relationship between four levels of objective greenness and perceived restorativeness in a sample of visitors to a large urban park in Milan, Italy (Parco Nord Milano—PNM).

We expect (a) stronger effects depending the amount of greenness (i.e., considering that the larger the buffer considered, the higher the amount of greenness), with connectedness towards nature as a confounder variable, on the dimensions of restorativeness; and (b) that the sunlight intensity would moderate the relationship between objective greenness and perceived restorativeness. More specifically, such a relationship would become stronger at higher level of sunlight.

## Materials and methods

### Participants and procedure

**Figure 1:**
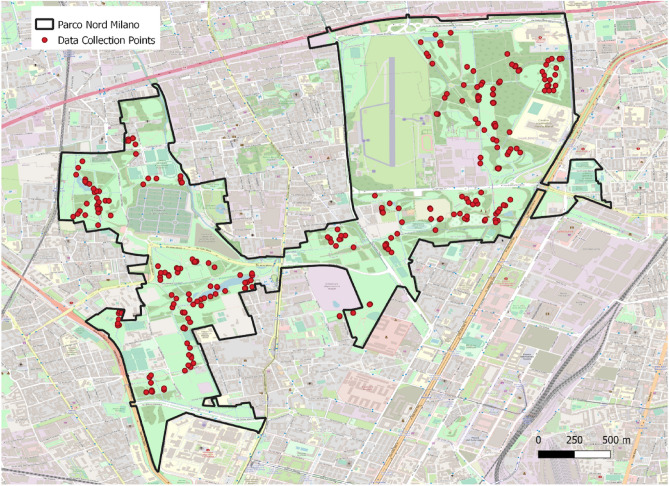
Map of data collection points in the study area i.e., Parco Nord Milan, Italy. Basemap retrieved from OpenStreetMap. https://www.openstreetmap.org/ (2020).

The present study included park visitors recruited during their visit at PNM. Convenience sampling technique was applied in order to reach the goal of at least ten visitors per selected data collection point in the park. Figure 1 shows the map of data collection points in the study area. Inclusion criteria to the study participation were: (a) being 18 years or older; (b) absence of cognitive impairment and/or mental or neurodegenerative illnesses. Park visitors were approached and invited to participate in our study research. We ensured each participant that the questionnaire was anonymous, and that the data would be processed in aggregate form. Furthermore, the possibility to withdraw their participation at any time was explained and a contact for questions and/or feedback was provided. Informed consent was obtained from all participants. A total of 312 participants gave their consent to participate in the study. Participants were interviewed individually by a well-trained interviewer in the same location where they had been approached. Data were collected through an online questionnaire. The interviewer was responsible for administering the questionnaire and recording participants’ responses using a tablet. Upon the completion of the questionnaire, the interviewer marked down the GPS coordinates of the exact location where the administration had been held and detected the temperature and the level of sunlight using a lux meter. The whole procedure had an average duration of approximately 30 min. The questionnaires were collected between the 23rd of June and the 28th of July 2021, both on weekdays and holidays. Data collection was carried out only during hours of daylight, i.e., in the morning (time slot: from 7:00 a.m. to 1:59 p.m.) and afternoon (time slot: from 2:00 p.m. until sunset), both to collect data on the presence of light as our variable of interest.

Recorded average temperature in July 2021 was 30.1 °C (min = 26.4 °C; max = 33.8 °C).

### Measures

#### Greenness

Greenness was assessed using Google Earth Engine^44^, a geospatial processing service developed by Google. As a data source, we used Sentinel-2 (European Spatial Agency) images for their fine spatial resolution (10 m) and wide temporal viability. First, we visually checked all the available Sentinel-2 images for our study area, which were acquired in the same time period of the interviewing procedure and had less than 10% of cloud cover. We selected one image acquired on the 25th of June 2021 (S2A_MSIL2A_20210625T102021_N0300_R065_T32TNR_20210625T132502), which was free of clouds over our study area. The image was already processed using *sen2cor* and provided at L2 level (surface reflectance) (Sentinel-2 User Handbook, 2015), hence no further radiometrically or atmospherically corrections were required. The selected image was used to calculate the Normalized Difference Vegetation Index (NDVI), a widely used greenness indicator^10^, as following:

$$ {\text{NDVI }} = \, \left( {{\text{B8 }}{-}{\text{ B4}}} \right)/ \, \left( {{\text{B8 }} + {\text{ B4}}} \right) $$

where *B8* and *B4* are the Sentinel-2 spectral bands capturing data from 0.785- to 0.899- μm (Near-Infrared) and 0.650- to 0.680-μm (Red) wavelength ranges, respectively. For each GPS coordinate position of the interviewed we extracted the NDVI pixel value and the mean NDVI values for 50-, 100-, 300- and 500-m circular buffers.

#### Sunlight intensity

The unit of measurement for estimating the magnitude of illuminance is lux (lx). The minimum threshold for distinguishing the forms present in an indoor environment is 20 lx while the maximum threshold for outdoor environments is 2000 lx. Sunlight intensity was measured using a digital light intensity meter for photography and indoor and outdoor lighting and temperature (detection range: 0.01–300,000 lx).

#### Perceived restorativeness

We refer to the ART^6^ in investigating the potential restorative quality of PNM. Perceived restorative quality of the environment was assessed using the Italian validation of the Perceived Restorativeness Scale—Short Version^45^ original validation by Hartig et al.^46^). The short version of the PRS scale has been validated in Italian and English. It is composed of 11 items belonging to four dimensions corresponding to as many sub-scales, i.e., the Fascination sub-scale (an example of item: “In places like this my attention is drawn to many interesting things”), the Being away sub-scale (an example of item: “To get away from things that usually demand my attention I like to go to places like this”), the Coherence sub-scale (an example of item: “In places like this everything seems to have its proper place”), and the Scope sub-scale (an example of item: “That place is large enough to allow exploration in many directions”). Ratings were made on a 5-point Likert scale from 1 (strongly disagree) to 5 (strongly agree). Authors of this scale did not report Alpha values, which was instead found to be satisfactory (i.e., 0.82) in our sample.

#### Connectedness to nature

Connectedness to nature was assessed using three items of the Love and Care for Nature (LCN) scale^47^, which operationalizes the construct of emotional connection (or “biophilia”) with nature (Cronbach’s Alpha = 0.97). The original 15-item scale is validated in English, and the chosen items were translated into Italian. For the sake of the brevity of the interview, given that it was administered to a sample of park visitors, and to maximize the possibility of having a large sample, we opted to use three items of the LCN scale as a measure of connectedness to nature. The items were chosen among those with the highest factor loadings reported in the original validation. Selected items, on a 7-point Likert scale ranging from 1 (strongly disagree) to 7 (strongly agree), were: “When I spend time in unspoilt nature, I feel that my day-to-day worries seem to dwindle away in the face of the wonder of nature” (factor loading in the original validation = 0.91) “I feel content and somehow at home when I am in unspoilt nature” (factor loading in the original validation = 0.90), and “I often feel emotionally close to nature” (factor loading in the original validation = 0.88).

#### Perceived park qualities

We investigated three variables regarding perceived park qualities using three items on a 5-point scale ranging from 1 (strongly disagree) to 5 (strongly agree). The three variables were: (a) thermal comfort; (b) air quality, and (c) noise reduction. Items were: “I come to the park to relieve the feeling of excessive heat”, “I come to the park to breathe clean air” and “At the park I find relief from the noise of the city”.

### Study area

Our study was based in a large urban area, (i.e., PNM 45° 53′ 71″ N, 9° 20′ 7″ E), located in the metropolitan area of Milan, in the region of Lombardy in Northern Italy. The site was chosen because of its potential benefits for human well-being that had already been documented in previous literature. For instance, Panno and collaborators^48^ reported a higher level of self-reported well-being and a lower level of depletion of cognitive resources among visitors of PNM during a particularly hot summer.

The area covers over a total of 790 ha and includes forest plantation, tree rows, agricultural areas, open spaces, infrastructures, and artificial areas, such as sports fields (see^49,50^) for detailed information on vegetation, microclimate, and soil characteristics). In order to represent the variety of green area characteristics within the entire park surface, 30 random points were randomly selected using the "Create Random Points" tool in ArcMap software, using the study area as a constraining extent (Fig. 1). For each point, a minimum of ten participants were retrieved. Park areas involved in the study were: (a) forest and agricultural areas, (b) lawns, (c) park with facilities.

### Statistical analyses

Preliminary analyses were conducted to explore the role of covariate variables on the PRS scores. A linear regression was used to investigate the effect of age and gender on PRS total score. To take account of different park qualities on the four subscales of PRS (i.e., Fascination, Being away, Coherence, and Scope), a one way ANOVA was performed using the park areas (i.e., forest and agricultural areas, lawns, and park with facilities) as grouping variables. The variable “time of the day” was splitted into two categories, i.e., “morning” and “afternoon”, according to the time when the interview was conducted. An independent sample t-test was used to assess differences in PRS total score using “time of day” as independent variable. In order to investigate the structural relationships between greenness (i.e., NDVI) and the four factors of perceived restorativeness, four Structural Equations Models (SEM), one for each greenness level (i.e., Model 1: NDVI buffer 50 m; Model 2: NDVI buffer 100 m, Model 3: buffer 300 m, Model 4: buffer 500 m), were performed using the *lavaan* package^51^ on the R software^52^. Each model was graphically reported using the *lavaanPlot* package. All the four examined models were composed of four latent variables (i.e*., Fascination, Being away, Coherence*, and *Scope*) based on the four factors of the 11-item PRS^45^ as outcomes. Each greenness level (i.e., observed variable) was separately used, as a first predictor, in each corresponding model. Connectedness to nature (i.e., latent variable), Ecosystem Services (i.e., latent variable), and gender were added in each model, as predictors, in the investigated relationship. Connectedness to nature was measured by three selected items of the LCN scale. Ecosystem services were measured by three items evaluating perceived park qualities, i.e., thermal comfort, air quality, and noise reduction. No ordinal or nominal variables were included in the models.

To assess the goodness of fit of the examined model the fit indices of the SEM were evaluated as follows (see Table 1): the Comparative Fit Index (CFI) which is considered as indicative of good fit if ≥ 0.9^53^, the Root Mean Square Residual (RMSEA) and the Standardized Root Mean Square Residual (SRMR), which are considered as suitable if < 0.08^54^. The Chi-squared value (c2) divided by the degree of freedom (c2/df), which indicates a good fit of the models if less than 5, was evaluated as well^55^.

**Table 1:** Descriptive statistics.

	Mean	SD
*N = 112*		
Age	52.9	20.9
Education	13.3	6.86
NDVI buffer 50 m	0.710	0.121
NDVI buffer 100 m	0.701	0.111
NDVI buffer 300 m	0.643	0.092
NDVI buffer 500 m	0.580	0.0741
Sunlight^a^	128	76
LCN	30.6	4.28
PRS Fascination	11.7	2.01
PRS Being Away	10.5	2.91
PRS Coherence	11.3	2.21
PRS Scope	7.96	1.42
PRS	41.4	6.04

*NDVI* normalized difference vegetation index, *LCN* love and care nature scale, *PRS* perceived restorativeness scale.

^a^Mean and SD of square root of sunlight intensity were reported.

In addition, sunlight intensity was used to evaluate its potential moderating effect in the relationship between greenness (i.e., NDVI, observed variables) and the total score of the PRS, obtained by summing the scores of the single items. Using the lavaan package^51^ on the R software^52^, four moderating models (i.e., one for each NDVI buffer) were estimated to investigate whether the association between different levels of greenness and the perceived restorativeness differs across different levels of sunlight intensity. Gender was included as a covariate. First of all, we used the square root transformation for sunlight intensity (i.e., observed variable). Then, we centred the interaction variables (i.e., NDVI at different buffers and sunlight intensity). A plot of the interaction effect was performed with the package interactions^56^ on the R software for each moderation model.

### Ethics declarations

The study was approved by the institutional ethics committee of the European University of Rome, Italy (protocol n. 06/2021). All methods were performed in accordance with the Declaration of Helsinki and its later amendments for studies involving human participants.

## Results

### Descriptive statistics

Descriptive statistics were shown in Table 1.

The sample was composed of 312 adults (43.6% women and 56.4% men) ranging between 18 and 91 years old (M = 52.9; DS = 20.9). Nearly all the participants were from Italy (90.4%), while the remaining were from other countries in Europe, Africa, and Asia (9.6%).

To explore the potential effects of demographic characteristics, i.e., age and gender, on total perceived Restorativeness, a linear regression analysis was performed. Association was found between gender and the total score of PRS (β = − 0.342) (Table S1). The 72.1% of the interviews were conducted in lawns areas, the 17.0% were conducted in forest and agricultural areas, and the 10.9% were conducted in the park with facilities areas. The one-way ANOVA revealed no differences between the three groups (Table S2).

Most of the interviews were conducted in the morning (64.1%). The variable “time of the day” was splitted into two categories, i.e., “morning” and “afternoon”, according to the time when the interview was conducted. An independent sample t-test did not reveal a statistically significant difference in PRS total scores between the two times of the day (Table S3).

Table 2 shows the correlations among variables; effect sizes of these relationship ranged from small (r = 0.116) to medium (r = 0.389).

**Table 2:** Correlations among variables involved in the models.

	NDVI buffer 50 m	NDVI buffer 100 m	NDVI buffer 300 m	NDVI buffer 500 m	LCN (selected item)	PRS fascination	PRS being away	PRS coherence	PRS scope	PRS	Thermal comfort	Air quality	Noise reduction
NDVI buffer 50 m	–												
NDVI buffer 100 m	0.878***	–											
NDVI buffer 300 m	0.418***	0.602***	–										
NDVI buffer 500 m	0.272***	0.379***	0.843***	–									
LCN (selected item)	− 0.077	− 0.052	0.052	0.118*	–								
PRS Fascination	0.050	0.097	0.118*	0.105	0.380***	–							
PRS Being Away	− 0.035	0.029	0.162**	0.131*	0.299***	0.385***	–						
PRS Coherence	0.106	0.116*	0.119*	0.130*	0.209***	0.424***	0.143*	–					
PRS Scope	0.048	0.095	0.130*	0.167**	0.219***	0.498***	0.228***	0.358***	–				
PRS	0.050	0.111	0.192***	0.185**	0.398***	0.790***	0.716***	0.660***	0.641***	–			
Thermal Comfort	0.201***	0.147**	0.053	0.047	0.182**	0.086	− 0.061	0.267***	0.020	0.101	–		
Air Quality	0.247***	0.198***	0.155**	0.144*	0.108	0.212***	0.074	0.086	0.194***	0.183**	0.262***	–	
Noise Reduction	0.096	0.121*	0.074	0.034	0.087	0.173**	0.094	0.191***	0.215***	0.223***	0.235***	0.130*	–

*NDVI* normalized difference vegetation index, *LCN* love and care nature scale, *PRS* perceived restorativeness scale.

*p* < 0.05, **p < 0.01, ***p < 0.001.

### Structural Equation Models

Taking into account different levels of greenness (i.e., NDVI buffer 50 m; NDVI buffer 100 m, NDVI buffer 300 m, NDVI buffer 500 m), four SEM models were performed to test our hypothesis. All examined models reported the NDVI score, at different buffers (as specified above) as predictor. The four factors of PRS as latent variables were used as outcomes (i.e., Fascination, Being away, Coherence, and Scope). To investigate the relationship between greenness levels and the four factors of perceived restorativeness, Connectedness to nature (i.e., latent variable) and of the Ecosystem services (i.e., latent variable), the latter were involved in the models as further predictors.

The fit indices indicated a goodness of fit for all four examined models (Table 3). In addition, in all models the factor loadings of the latent variables used as predictors (i.e., connectedness to nature and ecosystem service) and as outcomes (i.e., Fascination, Being away, Coherence, and Scope) were all statistically significant (*p* < 0.005).

**Table 3:** Fit indexes of structural equation models (SEM).

Model	CFI	RMSEA	SRMR	X^2^/df	AIC
Model 1 NDVI buffer 50 m	0.900	0.075	0.080	2.734	12,669.174
Model 2 NDVI buffer 100 m	0.900	0.073	0.078	2.641	12,665.875
Model 3 NDVI buffer 300 m	0.906	0.069	0.075	2.468	12,660.227
Model 4 NDVI buffer 500 m	0.904	0.069	0.077	2.501	12,661.402

*NDVI* normalized difference vegetation index, *CFI* comparative fit index, *TLI* Tucker-Lewis index, *RMSEA* root mean square error of approximation, *SRMR* standardized root mean square residual, *X*^*2*^ Chi –square, *df* degrees of freedom, *AIC* akaike.

**Figure 2:**
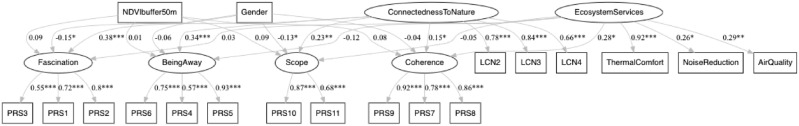
Structural equation model examining the effect of NDVI 50 m buffer, connectedness to nature (latent variable), ecosystem services (latent variable), and gender on fascination, being away, coherence and scope (latent variable). *Note* NDVI = Normalized Difference Vegetation Index; LCN = Love and Care Nature Scale; PRS = Perceived Restorativeness Scale.

*SEM Model 1 NDVI buffer 50 m* (Fig. 2) The SEM results for the first model revealed that NDVI buffer 50 m was not significantly associated with Fascination, Being away, Coherence and Scope (Table 4). The analysis showed that the effect of Connectedness to nature was significantly associated with Fascination (β = 0.0.375), Being away (β = 0.342), Coherence (β = 0.146) and Scope (β = 0.225) (Table 4). No statistically significant associations were found between ecosystem services and outcomes, except for coherence (β = 0.281). Associations were found between gender and Fascination (β = − 0.148) and Scope (β = − 0.133).

**Table 4:** Effect of NDVI at different buffers, connectedness to nature and ecosystem service on the four factors of perceived restorativeness.

Model	Effect	Standardized estimate	p value	Effect	Standardized estimate	*p* value	Effect	Standardized estimate	*p* value	Effect	Standardized estimate	*p* value
Model 1 NDVI buffer 50 m	*Of NDVI buffer 50 m*			*Of individual propensity to nature*			*Of ecosystem service*			*Of gender*		
	On fascination	0.094	0.119	On fascination	0.375	0.000**	On fascination	0.033	0.633	On fascination	− 0.148	0.014*
	On being away	0.015	0.795	On being away	0.342	0.000***	On being away	− 0.115	0.084	On being Away	− 0.058	0.311
	On coherence	0.079	0.158	On coherence	0.146	0.026*	On coherence	0.281	0.000***	On coherence	− 0.035	0.534
	On scope	0.093	0.131	On scope	0.225	0.000**	On scope	− 0.048	0.491	On scope	− 0.133	0.030*
Model 2 NDVI buffer 100 m	*Of NDVI buffer 100 m*			*Of individual propensity to nature*			*Of ecosystem service*			*Of gender*		
	On Fascination	0.142	0.018*	On Fascination	0.373	0.000***	On fascination	0.036	0.602	On fascination	− 0.145	0.015*
	On Being Away	0.046	0.422	On Being Away	0.344	0.000***	On being away	− 0.120	0.075	On being away	− 0.057	0.320
	On Coherence	0.084	0.135	On Coherence	0.140	0.032*	On coherence	0.290	0.000***	On coherence	− 0.033	0.557
	On Scope	0.131	0.032*	On Scope	0.223	0.001***	On scope	− 0.044	0.528	On scope	− 0.130	0.034*
Model 3 NDVI buffer 300 m	*Of NDVI buffer 300 m*			*Of individual propensity to nature*			*Of ecosystem service*			*Of gender*		
	On fascination	0.127	0.036*	On fascination	0.346	0.000***	On fascination	0.089	0.245	On fascination	− 0.139	0.021*
	On being away	0.160	0.004**	On being away	0.337	0.000***	On being away	− 0.125	0.088	On being away	− 0.050	0.382
	On coherence	0.107	0.055	On coherence	0.109	0.101	On coherence	0.337	0.000***	On coherence	− 0.030	0.595
	On scope	0.131	0.033*	On scope	0.199	0.004**	On scope	0.009	0.913	On scope	− 0.122	0.048*
Model 4 NDVI buffer 500 m	*Of NDVI buffer 500 m*			*Of individual propensity to nature*			*Of ecosystem service*			*Of gender*		
	On fascination	0.079	0.197	On fascination	0.337	0.000***	On fascination	0.109	0.173	On fascination	− 0.144	0.017*
	On being away	0.128	0.024*	On being away	0.323	0.000***	On being away	− 0.117	0.120	On being away	− 0.058	0.312
	On coherence	0.125	0.025*	On coherence	0.088	0.189	On coherence	0.352	0.000***	On coherence	− 0.036	0.522
	On scope	0.139	0.024*	On scope	0.182	0.010*	On scope	0.028	0.729	On scope	− 0.126	0.042*

*NDVI* normalized difference vegetation index

*p* < 0.05, **p < 0.01, ***p < 0.001.

**Figure 3:**
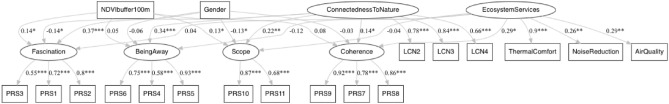
Structural equation model examining the effect of NDVI 100 m buffer, connectedness to nature (latent variable), ecosystem services (latent variable), and gender on fascination, being away, coherence and scope (latent variable). *Note* NDVI = Normalized Difference Vegetation Index; LCN = Love and Care Nature Scale; PRS = Perceived Restorativeness Scale.

*SEM Model 2 NDVI buffer 100 m* (Fig. 3) The SEM results for the second model showed that the NDVI buffer 100 m was significantly associated with Fascination (β = 0.142) and Scope (β = 0.131) (Table 4). NDVI buffer 100 m was not significantly associated with Being away and Coherence (Table 4). The analysis showed that the effect of Connectedness to nature was significantly associated with Fascination (β = 0.0.373), Being away (β = 0.344), Coherence (β = 0.140) and Scope (β = 0.223) (Table 4). No statistically significant associations were found between ecosystem services and outcomes, except for coherence (β = 0.290). Associations were found between gender and Fascination (β = − 0.145) and Scope (β = − 0.130).

**Figure 4:**
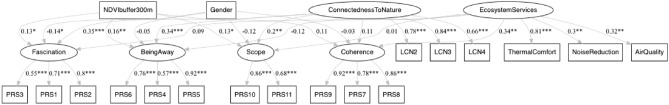
Structural equation model examining the effect of NDVI 300 m buffer, connectedness to nature (latent variable), ecosystem services (latent variable), and gender on fascination, being away, coherence and scope (latent variable). *Note* NDVI = Normalized Difference Vegetation Index; LCN = Love and Care Nature Scale; PRS = Perceived Restorativeness Scale.

*SEM Model 3 NDVI buffer 300 m* (Fig. 4) The SEM results for the third model indicated that the NDVI buffer 300 m was significantly associated with Fascination (β = 0.127), Being away (β = 0.160), and Scope (β = 0.131) (Table 3). NDVI buffer 300 m was not significantly associated with Coherence (Table 4). Connectedness to nature was significantly associated with Fascination (β = 0.346), Being away (β = 0.337), and Scope (β = 0.199) (Table 3) while it was not with Coherence (Table 4). No statistically significant associations were found between ecosystem services and outcomes, except for coherence (β = 0.337). Associations were found between gender and Fascination (β = − 0.139) and Scope (β = − 0.122).

**Figure 5:**
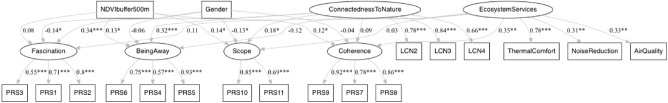
Structural equation model examining the effect of NDVI 500 m buffer, connectedness to nature (latent variable), ecosystem services (latent variable), and gender on fascination, being away, coherence and scope (latent variable). *Note* NDVI = Normalized Difference Vegetation Index; LCN = Love and Care Nature Scale; PRS = Perceived Restorativeness Scale.

*SEM Model 4 NDVI buffer 500 m* (Fig. 5) The SEM results for the fourth model indicated that the NDVI buffer 500 m was not statistically associated with Fascination (Table 4). The effect of NDVI buffer 500 m was statistically significant on Being away (β = 0.128), Coherence (β = 0.125), and Scope (β = 0.139) (Table 4). Connectedness to nature was not significantly associated with Coherence; on the contrary Ecosystem services was found to be associated with Coherence (β = 0.352) but not with the other outcomes. Associations were found between gender and Fascination (β = − 0.144) and Scope (β = − 0.126).

### Moderation models

**Figure 6:**
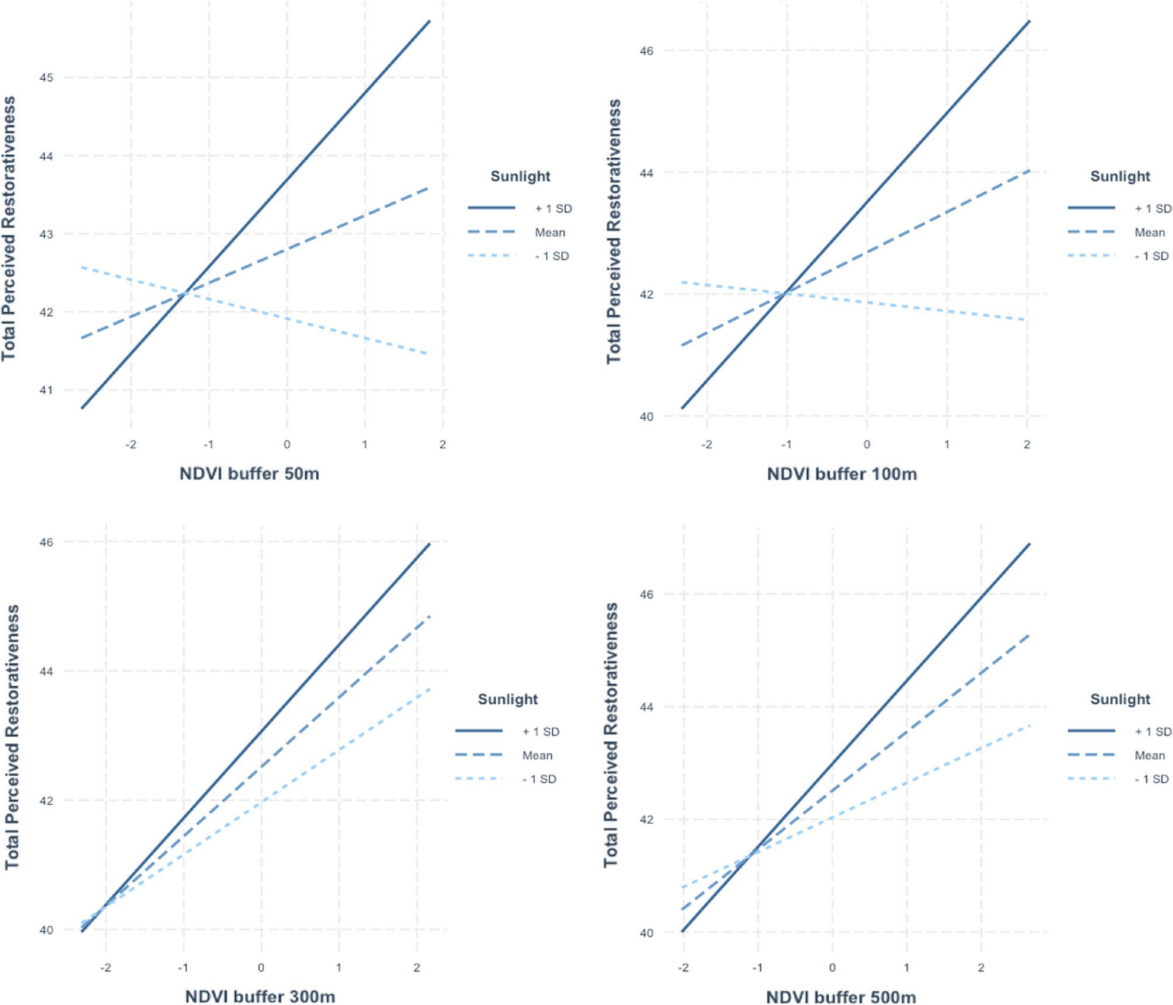
Interaction plots of moderation analyses. *Note*: NDVI = Normalized Difference Vegetation Index.

*Moderation Model 1 NDVI buffer 50 m* The effect of NDVI buffer 50 m and total perceived restorativeness were not found, but a significant positive effect was found between Sunlight intensity and total Perceived restorativeness (β = 0.164). Gender was revealed to be negatively associated with total Perceived restorativeness (β = − 0.181), with women reporting less restorativeness than men. A significant interaction effect of NDVI buffer 50 m and Sunlight intensity on total Perceived restorativeness was found (β = 0.110). The conditional effects were estimated respectively adding and subtracting to the Sunlight sample mean one standard deviation. Two Sunlight levels were obtained. For high levels, Sunlight was found to be a significant moderator (β = 0.182), whereas for low levels it was not found to be significant (β = − 0.038) (Fig. 6).

*Moderation Model 2 NDVI buffer 100 m* A significant positive effect was found between NDVI buffer 100 m (β = 0.110) and Sunlight intensity (β = 0.150) on total Perceived restorativeness, with women reporting less restorativeness than men. Gender was revealed to be negatively associated with total Perceived restorativeness (β = − 0.174). The interaction effect of NDVI buffer 100 m and Sunlight intensity on total Perceived restorativeness was not significant (Fig. 6).

*Moderation Model 3 NDVI buffer 300 m* A significant positive effect was found for NDVI buffer 300 m (β = 0.171) and Sunlight intensity (β = 0.108) on total Perceived restorativeness. Gender was revealed to be negatively associated with total Perceived restorativeness (β = − 0.171), with women reporting less restorativeness than men. Interaction effect of NDVI buffer 300 m and Sunlight intensity on total Perceived restorativeness was not significant (Fig. 6).

*Moderation Model 4 NDVI buffer 500 m* A significant positive effect was found for NDVI buffer 500 m on total Perceived restorativeness (β = 0.165). Gender was revealed to be negatively associated with total Perceived restorativeness (β = − 0.175), with women reporting less restorativeness than men. Significant effect was not found for Sunlight on total Perceived restorativeness. No significant interaction effect of NDVI buffer 500 m and Sunlight intensity on total Perceived restorativeness was found (Fig. 6).

## Discussion

The present work was intended to provide insights on the effect of objective environmental measures, such as greenness level and sunlight intensity, on the four dimensions of the perceived restorativeness in a sample of park visitors, taking into account the role of the personal attitudes towards nature.

Altogether, we observed differentiated effects on the dimensions of restorativeness in relation to the distance within which greenness level is measured. Connectedness to nature confirmed previous evidence on its role in predicting dimensions of restorativeness, i.e., fascination, scope, and being away^21^. Our findings confirmed our first hypothesis that the amount of greenness (i.e., considering that the larger the buffer considered, the higher the amount of greenness) positively impact in a differentiated way on dimensions of restorativeness. Greenness level with a buffer size at 300 m was associated with several dimensions of restorativeness, i.e., Fascination, Scope, and Being away. Consistently, greenness level with a buffer size at 500 m was associated with Coherence, Scope, and Being Away. When an individual is surrounded by an green space of at least 300 m, he/she would benefit from certain dimensions related to the sense of restorativeness. We might speculate that 300 m is the minimum distance able to create the perceptual condition for a sense of immersion in nature. It is worth mentioning that epidemiological evidence suggested that a distance of 300 m from the nearest park or green space, i.e., residential surrounding greenness, is significantly associated with better mental health, less medication use, fewer psychologist, or psychiatrist visits^57^ and a number of mental and cognitive health outcomes, e.g., attention in children^58^, geriatric depression^59^, depressive symptoms in pregnant women^60^. However, the aforementioned studies considered the amount of greenness within a built area, for example in a neighborhood, thus not considering the potential intrusive effect of the gray, built features within that area.

Nature exposure, as in the case of PNM, contributes to existing evidence relating nature immersion to higher perceived well-being^61^.

Previous findings on Italian samples showed that perceived restorativeness did not differ for age and gender^62–64^. Our results based on the exploration of each PRS dimension confirmed those findings regarding age, while gender was found to be associated with PRS dimensions of Fascination and Scope. Gender was also found to be significant in the moderation models, with women reporting less perceived restorativeness than men. The role of gender deserves attention since, despite accumulating evidence showed that women are more engaged in pro-environmental behaviors, men might perceive stronger benefits in interacting with nature, as observed in our results. It would be useful to explore why, in the face of greater perceived well-being, men are generally less engaged in activities that include the preservation and promotion of a sustainable environment.

Interestingly, specific park qualities, e.g., thermal comfort, air quality, and noise reduction, were found to be associated with the PRS dimension of Coherence. Previous results reported the link between perception of reduced noise, increased air quality and thermal comfort, and olfactory pleasantness, as ecosystem services provided by urban parks, and perceived restorativeness and well-being^21,48,65–68^.

The novelty lies in the detection of the dimension associated with them, i.e., Coherence. We can assume that ecosystem services are perceived as referring to structural dimensions, such as the park design. As previously mentioned, Coherence concerns the relationships between objects present in the external world; on the contrary, the other three PRS dimensions concerns the internal world of the visitors within an environment.

Our preliminary finding on the interaction effect of buffer level and sunlight showed that the lower buffer level is associated with perceived restorativeness only in case of higher level of sunlight intensity. We could infer that the level of sunlight may enhance the positive effect of greenness only at certain buffer distances. At the same time, we cannot exclude that the clarify of direct sunlight attenuates at greater distance, reducing color saturation, so that its effect is only perceived within a 50 m buffer, and its positive effect on restorativeness is limited by that. Further examinations are needed, however, as preliminary as this result is, it taps into a less-investigated research line that explores not only the role of indoor light, for example in workplaces, hospitals, and schools, but also that of daylight in open spaces^38^. It would be appropriate to supplement these results by investigating potential effects of brightness and shading in conjunction with other important variables, such as outdoor temperature and seasonality.

On this subject, a remarkable point is that urban parks confirmed their protective role for visitors’ perceived well-being during periods at high risk of heatwaves, in line with the hypothesis that the visit and use of urban green spaces could alleviate the perception of thermal discomfort in areas where episodes of heat-related stress are more likely to occur^48,67^.

Additionally, our study highlights that there is room for improvement to further investigate both the role of individual and environmental/ecological variables. For example, it is important to understand how users use their time within the park (e.g., physical activity, social interactions), frequency of visits, and the amount of time spent per visit, in light of recent findings reporting that exposure to at least 120 min of nature is associated with health and well-being^69^. It would also be worth exploring the restoration needs of park visitors, as a crucial aspect in affecting how they judge the restorative quality of an environment^70^ and the level of familiarity with public green spaces and individual differences, e.g., in the elderly, healthy or clinical, population^71^.

It also would be useful exploring other objective measures, for example related to shading (e.g., canopy cover) to disentangle the relationship between greenness exposure, lighting, temperature effects and perceived well-being.

Our study attempted to introduce the use of objective measure of greenspace on perceived psychological variables and for this reason we started by testing the gold standard measure, i.e., the NDVI. For future studies, we plan to extract and test other indices on the variables of our interest. For this purpose, employing more complex indicators for space characterization, by moving from a two- to a three-dimensional approach to consider the combination between green and built-up elements in heterogeneous urban contexts is warranted^72^.

## Conclusions

In light of the current global challenges, including growing urbanization, recent pandemic-related burdens, and climate change threats, evidence is accumulating on the crucial role of urban green space in mitigating detrimental effects of these scenarios on health and well-being. Added to this is the growing awareness among citizens of the importance of parks and green space in general as a social, recreational, and therapeutic place, especially in large cities like Milan with highly centralized greenspace where epidemiological studies relating residential distance to greenspace are not very transferable. In these cases, in fact, urban planning should be tailored to citizens, both to increase the attractiveness of urban green spaces, and therefore the probability of contact with urban nature, and to foster their potential benefits. This is only possible by exploring the complexity of relationships among natural elements, types of landscapes, and user needs.

Our work is intended to be exploratory and preliminary of a growing research topic. The underlying hypothesis was that even when an individual stands still in a place for a while, he/she may benefit from nature exposure. The short-term exposure is given by the stay in a green area (i.e., a park, in this case) for a minimum time equally to the time spent for the interview (approximately 30 min). Future studies could explore what the minimum amount of green exposure time is required to have a perceived effect on well-being.

### Supplementary Information

Supplementary Tables.

## Data Availability

The datasets generated during and/or analysed during the current study are available from the corresponding author on reasonable request.
