# Supplementary material for: Objective greenness, connectedness to nature and sunlight levels towards perceived restorativeness in urban nature
Source: Sci Rep. 2023 Oct 24;13:18192. doi: 10.1038/s41598-023-45604-3 (PMC10598040; doi:10.1038/s41598-023-45604-3)
Supplement: Supplementary file 1 — Supplementary Tables. [file 41598_2023_45604_MOESM1_ESM.pdf]

Table S1

Linear regression on the PRS total score with age and gender as independent variables.

	Estimate	p
**Age**	-0.082	0.149
**Gender**	-0. 342	0.003

Table S2

One-way ANOVA on the four PRS subscales with park areas as grouping variable.

	Forest and Agricultural fields	Lawns	Park with facilities	p
**PRS Fascination**	12.00±1.81	11,76±2.00	11.13±2.12	0.075
**PRS Being Away**	10.71±2.92	10.39±2.92	10.60±2.91	0.774
**PRS Coherence**	10.53±2.26	11.38±2.17	11.19±2.29	0.110
**PRS Scope**	40.91±5.51	41.57±5.96	40.72±6.71	0.237

Note: PRS= Perceived Restorativeness Scale

Table S3

Independent sample t- test on PRS total score with time of day as grouping variable.

	Morning	Afternoon	p
**PRS**	41.5 ± 6.39	41.1±5.39	0.633

Note: PRS= Perceived Restorativeness Scale
